# High Dose Pharmaceutical Grade Biotin (MD1003) Accelerates Differentiation of Murine and Grafted Human Oligodendrocyte Progenitor Cells In Vivo

**DOI:** 10.3390/ijms232415733

**Published:** 2022-12-12

**Authors:** Marion J. F. Levy, Beatriz Garcia-Diaz, Frédéric Sedel, Anne Baron-Van Evercooren, Sabah Mozafari

**Affiliations:** 1 INSERM, U1127, 75013 Paris, France; 2 CNRS, UMR 7225, 75013 Paris, France; 3 Faculty of Medicine, Sorbonne Université, UPMC Paris 06, UM-75, 75005 Paris, France; 4 GH Pitié-Salpêtrière, Institut du Cerveau (ICM), 75013 Paris, France; 5 MedDay Pharmaceuticals SA, 75008 Paris, France; 6 Laboratoire Matière et Système Complexe, MSC-Med, CNRS, UMR 7057, Université de Paris Cité, 75006 Paris, France

**Keywords:** MD1003, murine oligodendrocytes, human oligodendrocytes, humanized animal model, shiverer mice, oligodendrocyte differentiation, myelination

## Abstract

Accumulating evidences suggest a strong correlation between metabolic changes and neurodegeneration in CNS demyelinating diseases such as multiple sclerosis (MS). Biotin, an essential cofactor for five carboxylases, is expressed by oligodendrocytes and involved in fatty acid synthesis and energy production. The metabolic effect of biotin or high-dose-biotin (MD1003) has been reported on rodent oligodendrocytes in vitro, and in neurodegenerative or demyelinating animal models. However, clinical studies, showed mild or no beneficial effect of MD1003 in amyotrophic lateral sclerosis (ALS) or MS. Here, we took advantage of a mouse model of myelin deficiency to study the effects of MD1003 on the behavior of murine and grafted human oligodendrocytes in vivo. We show that MD1003 increases the number and the differentiation potential of endogenous murine oligodendroglia over time. Moreover, the levels of MD1003 are increased in the plasma and brain of pups born to treated mothers, indicating that MD1003 can pass through the mother’s milk. The histological analysis of the grafted animals shows that MD1003 increased proliferation and accelerates differentiation of human oligodendroglia, but without enhancing their myelination potential. These findings provide important insights into the role of MD1003 on murine and human oligodendrocyte maturation/myelination that may explain the mitigated outcome of ALS/MS clinical trials.

## 1. Introduction

Biotin is a water-soluble vitamin B-complex and an essential cofactor for five carboxylases involved in various cellular metabolic pathways including gluconeogenesis, fatty acid synthesis, metabolism of amino acids and fatty acids [1]. Studies have reported that nutritional biotin deficiency or genetic defects in biotinidase (an enzyme that helps recycle biotin) can induce immunological disorders, metabolic abnormalities, seizures, [2,3], demyelination and neurodegeneration [4,5,6].

During central nervous system (CNS) development, oligodendrocyte progenitor cells (OPCs) migrate into—and colonize—the developing white matter [7,8], where they undergo differentiation into mature oligodendrocytes by expressing a subset of myelin-associated proteins [9]. In addition to their contributions to neuronal signaling, oligodendrocytes maintain axonal integrity and provide trophic and metabolic support to neurons [10,11,12].

Myelin disorders, as a group, are among the most prevalent and disabling neurological conditions with no cure. In CNS demyelinating diseases, such as multiple sclerosis (MS), although remyelination occurs, it often fails due to (i) a primary deficiency in OPC numbers; (ii) a failure of their recruitment, (iii) differentiation or (iv) full maturation into myelin-forming cells [13,14]. Two main approaches have been proposed to enhance remyelination [15]. The first approach involves stimulation of endogenous OPCs to form mature myelinating oligodendrocytes [16,17] and the second one, provides a new pool of myelin-competent cells via transplantation [18,19,20].

Different agents have been studied in vitro or in animal models aiming to enhance endogenous repair [21]. Metabolic pathways have been suggested as possible therapeutic targets for progressive MS [22]. At the preclinical level, biotin enrichment in rodent oligodendrocytes was first described by LeVine and Macklin [23]. It was also found that biotin attenuates oxidative stress, mitochondrial dysfunction, lipid metabolism alteration and 7β-hydroxycholesterol-induced cell death in 158N murine oligodendrocytes [24], and might promote myelin synthesis by enhancing fatty acid production and increasing energy production by acting as a coenzyme in oligodendrocytes and neurons [25,26]. High-dose pharmaceutical grade biotin (MD1003) restores redox balance, energy and lipid homeostasis, and axonal health in a model of adrenoleukodystrophy [27]. Finally, biotin promotes survival, ensheathment and ATP production by rat oligodendrocyte lineage cells in vitro [28]. At the clinical level, safety and efficacy of MD1003 were evaluated in ALS patients [29]. Initial clinical data showed that daily doses of MD1003 up to 300 mg over 12 months, can improve objective measures of MS-related disability [30]. Collongues and collaborators reported that MD1003 treatment was associated with clinical as well as brain and cervical spinal cord volume improvements after one year of treatment [31]. However, the more comprehensive SPI2 study showed that MD1003 did not significantly ameliorate disability or walking speed in patients with progressive MS [32]. In spite of such paradoxical reports, a direct effect of MD1003 on the competence of oligodendroglial cells in vivo remains unclear. Here, we took advantage of a mouse model of myelin deficiency to study the effects of MD1003 on the behavior of both murine and grafted human oligodendrocytes in vivo. We show that although MD1003 can accelerate the differentiation potential of both murine and human oligodendrocytes in vivo, it does not increase the myelination potential of mature oligodendrocytes.

## 2. Results

### 2.1. MD1003 Increases the Number and Differentiation Potential of Murine OPCs In Vivo

To study the effect of MD1003 on the behavior of murine oligodendrocytes, myelin-deficient *Shi/Shi:Rag2^−/−^* mice (hereafter named shiverer) were fed with either MD1003 or vehicle pellets and analyzed over periods of 12, 16 and 20 weeks post treatment (wpt). While food consumption was regular and similar for all groups of mice, they did not show any sign of morbidity, mortality or any clinical sign of worsening of their “shiverer” phenotype (shivering and convulsion).

**Figure 1:**
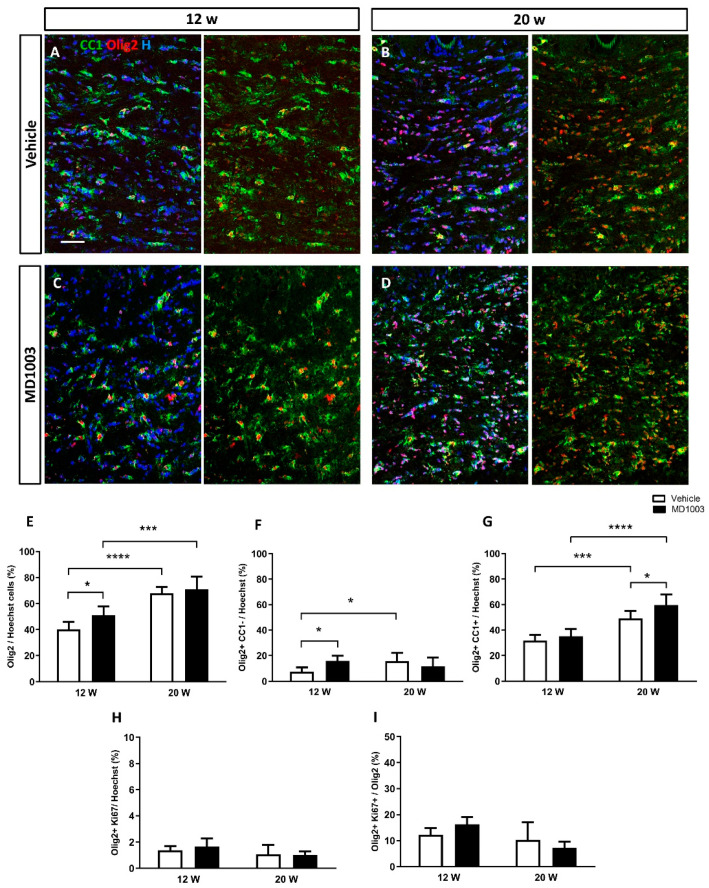
MD1003 accelerates the differentiation potential of murine OPCs in vivo. (**A**–**E**) The percentage of the total Olig2+ cells (red) over Hoechst (**H** in blue) increases over time for both vehicle- and MD1003-treated groups. MD1003 significantly increases the number of murine OPC at 12 wpt (**F**) and the OPC differentiation potential (CC1+ in green) at 20 wtp (**G**) in the brain of shiverer mice. There is no effect on the percentage of Ki67+OLIG2+/Hoechst+ cells (**H**) or Ki67+OLIG2+/OLIG2+ cells (**I**). Two-way ANOVA followed by the Tukey’s multiple comparison test (*n* = 5–6 mice per group). * *p <* 0.05, and *** *p <* 0.001, **** *p <* 0.0001. Error bars represent SEMs. WPT: weeks post-treatment; scale bar: 50 µm.

The effect of treatment was first analyzed by immunohistochemistry using OLIG2, a general oligodendroglial marker, and CC1, a marker of mature oligodendrocytes (OLs) (Figure 1A–D). Results showed a significant increase over time in the percentage of OLIG2+ cells in both vehicle and MD1003 groups (vehicle: 40.28 ± 2.19 and 67.86 ± 1.89 at 12 wpt and 20 wpt, respectively (*p* value = 0.0001); MD1003: 50.97 ± 2.56% and 71.16 ± 3.87% at 12 wpt and 20 wpt, respectively (*p* value = 0.0001)) (Figure 1E). In addition, MD1003 treatment significantly promoted the percentage of murine OPCs (OLIG2+/CC1-) at 12 wpt (vehicle: 7.35 ± 1.29% vs. MD1003: 15.88 ± 1.48%, *p* value = 0.031) (Figure 1F), as well as the percentage of murine OLs (OLIG2+/CC1+) at the later time-point of 20 wpt (vehicle: 49.14 ± 2.20 vs. MD1003: 59.62 ± 3.4, *p* value = 0.027) (Figure 1G). Thus, the increase in OLIG2+ cells resulted from an increase in OPC at 12 wpt, while that at 20 wpt resulted from an increase in murine OLs, suggesting an effect of MD1003 on two different stages of the lineage. To gain insight into the mechanism by which MD1003 acts on OLIG2+ cell numbers, we examined the effect of the drug on murine OLIG2+ cell proliferation. Immunolabeling for OLIG2 and the proliferation marker KI67 showed no difference in the percentage of OLIG2+KI67+ cells over the total number of cells (vehicle: 1.37 ± 0.16% vs. MD1003: 1.66 ± 0.35% at 12 wpt and vehicle: 1.06 ± 0.35% vs. MD1003: 1 ± 0.14% at 20 wpt), nor in the percentage of OLIG2+KI67+ cells over OLIG2+ cells (vehicle: 12.32 ± 2.58% vs. MD1003: 16.31 ± 2.8% at 12 wpt and vehicle: 10.31 ± 6.67% vs. MD1003: 7.29 ± 2.32% at 20 wpt) (Figure 1H,I).

**Figure 2:**
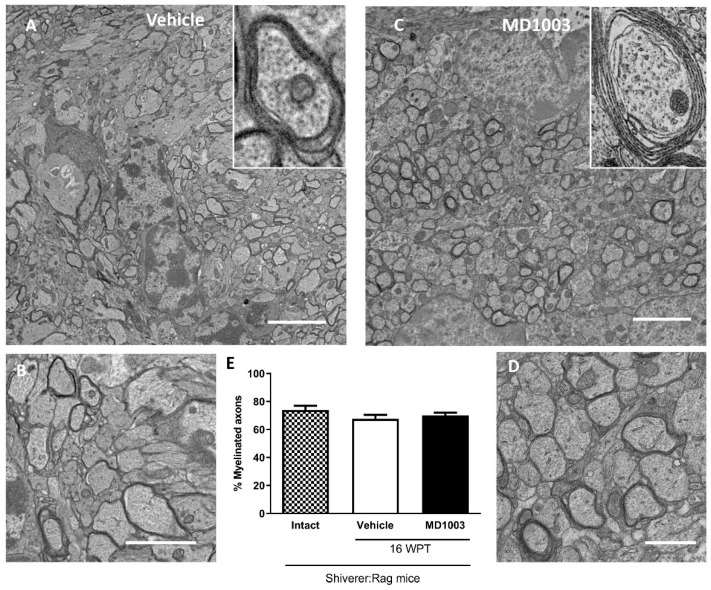
Electron microscopy evaluation of the percentage of myelinated axons by murine OLs in dysmyelinated shiverer mice. (**A**–**D**) Ultrastructure of endogenous uncompacted myelin in sagittal sections of the core of the corpus callosum 16 wpt with vehicle (**A**,**B**) and MD1003 (**C**,**D**). (**B**,**D**) and insets, are higher magnifications of (**A**,**C**). (**E**) Quantification of the percentage of myelinated axons revealed no significant difference between the vehicle, intact (not treated) and MD1003-treated mice. Two-way ANOVA was followed by the Tukey’s multiple comparison test (*n* = 3 mice per group). Error bars represent SEMs. WPT: weeks post-treatment; scale bars in (**A**,**C**): 5 μm, (**B**): 2 µm and (**D**): 1 µm.

The myelination potential of murine OLs was evaluated by electron microscopy and the percentage of myelinated axons in shiverer mice treated with MD1003 or vehicle for 16 weeks was quantified (Figure 2A–D). Shiverer mice, due to the absence of MBP, are characterized by fewer myelinated axons, and uncompacted myelin at the ultrastructural level, compared to wild-type mice [20,33,34]. Our data showed no significant difference in the percentage of myelinated axons between the treatment (70.13%) and vehicle (67.61%, *p* value = 0.7824) or intact (no treatment) groups (73.98%, *p* value = 0.5804), suggesting that MD1003 did not increase the percentage of myelinated axons by murine OLs at this time-point (Figure 2E).

### 2.2. MD1003 Accelerates the Differentiation of Human OPCs into Mature Oligodendrocytes In Vivo

**Figure 3:**
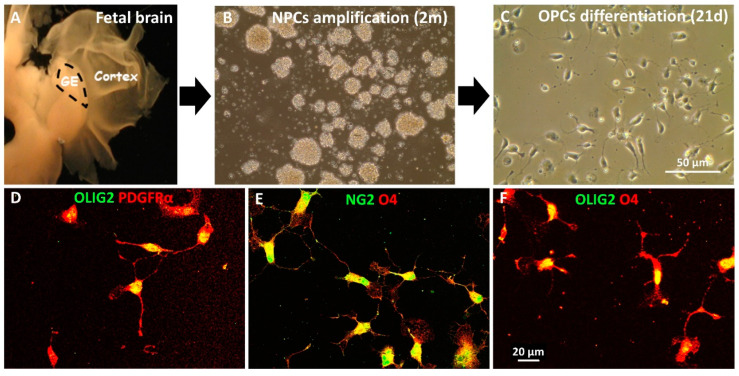
In vitro differentiation of hNPCs into hOPCs. (**A**) hNPC isolated from ganglionic eminence (GE) of the fetal brain were expanded as neurospheres in vitro for two months (**B**) before inducing them to differentiate into OPCs for 21 days (**C**). (**D**–**F**) Immunocytochemical characterization of the hOPCs illustrates their bipolar phenotype and expression of early stage oligodendroglial markers PDGFRα (red), NG2 (green) and O4 (red) in addition to OLIG2 (green).

Next, we investigated the effect of MD1003 on the behavior of human oligodendrocytes in vivo. For this purpose, we used fetal human neural precursor cells (hNPCs). The hNPCs were characterized previously as Nestin+ cells, not expressing mature markers of somatic neural cells [35]. Three fetal hNPC lines derived from ganglionic eminence (GE), were selected for OPC differentiation based on their marker expression, speed of growth and previous validation as lines capable of myelinating axons after engraftment in the developing murine brain (Figure 3A) [35,36]. After cell expansion in vitro (Figure 3B), cells were induced to differentiate into OPC using an in-house protocol [36]. After three weeks in differentiation medium, cells acquired a bipolar OPC-like phenotype (Figure 3C). In addition, immunocytochemistry showed that all three lines expressed the OPC markers PDGFR-α, NG2 and the late OPC marker, O4 (Figure 3). Immunocytochemistry for the general oligodendroglial markers SOX10 and OLIG2, and the OPC marker PDGFR-α, confirmed their enrichment into immature stages of the oligodendroglial lineage with 72.57 ± 2.17% of OLIG2+ cells, 84.56 ± 1.74% of SOX10+ cells, 70.52 ± 2.88% of PDGFRα+ cells.

Next, we took advantage of a humanized mouse model of dysmyelination to investigate the effect of MD1003 on the behavior of grafted human oligodendroglia in vivo. The *Shi/Shi:Rag2^−/−^* mice are immune- and MBP-deficient [20,34]. The immune deficient genotype avoids rejection of the grafted human cells. Moreover, any detectable MBP+ myelin by immunohistochemistry, or compact myelin by electron microscopy in these mutants validates that myelin derives from the wild-type donor cells [37]. Human donor cells can also be identified by the human-specific STEM markers and their stage of differentiation combining OLIG2 and CC1 markers: STEM+/OLIG2+/CC1- are hOPCs and STEM+/OLIG2+/CC1+ cells are mature hOLs.

hOPCs were transplanted in the corpus callosum of the shiverer pups at P1 while their mothers were under MD1003 or vehicle treatment as of postnatal day 0 (P0). To ensure MD1003 effective exposure to the pups early after engraftment, bio-distribution analysis was performed to measure MD1003 concentration in the plasma and the brains of P14 pups and their mothers using HPLC (CiToxLAB, Évreux, France). The presence of MD1003 was below detection level in the plasma and brains of the vehicle group (Table 1). However, detectable MD1003 concentrations were observed in both plasma and brains of the pups and their treated mothers, thus showing that MD1003 can pass through milk to increase plasma and brain levels of P14 pups as high as those of their mothers with a 20-fold increase in the brains of MD1003-treated over vehicle-treated groups (Table 1).

We and others previously reported that engraftment of hOPC increased shiverer mice survival [36,38]. While we did not develop an experimental paradigm to follow large cohorts until death to address this issue, no effect on mice survival was observed among the different experimental groups. Grafted animals were sacrificed at 12- and 20-week post-graft (wpg) for immunohistochemistry, and at 20 wpg for electron microscopy.

**Figure 4:**
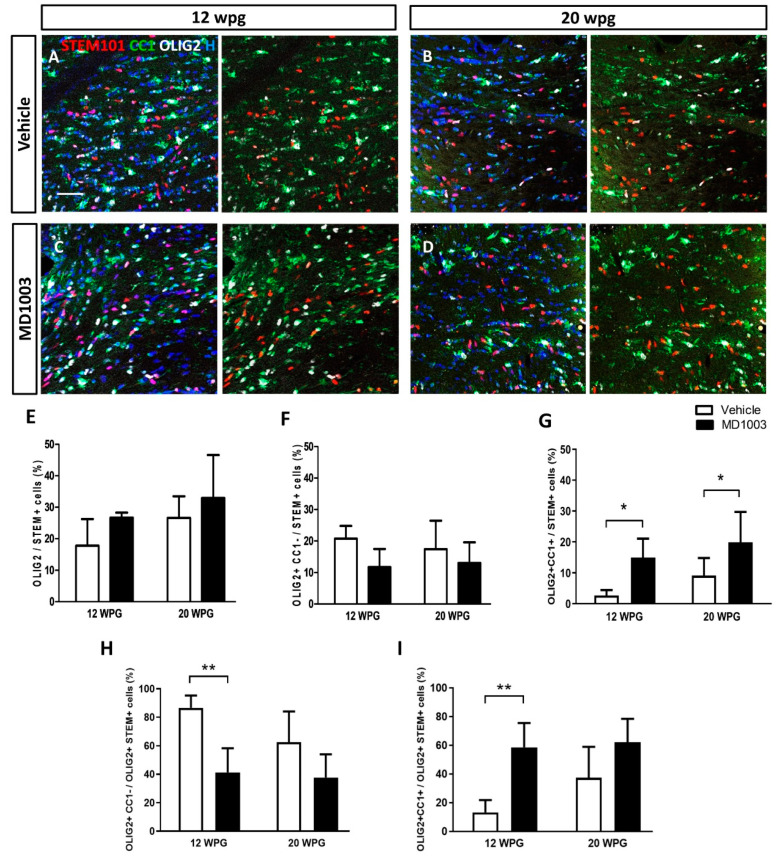
MD1003 increases the percentage of differentiated hOPCs. (**A**–**D**) STEM101+ human cells (red) are found in all the grafted animals 12 wpg or up to 20 wpg. (**E**) The percentage of total hOLs (OLIG2, in white) tends to increase over time, and in MD1003- vs. vehicle-treated animals. (**F**) While the percentage of OLIG2+CC1- hOPCs (CC1 in green) tends to slightly decrease overtime, MD1003-treated mice contain significantly higher numbers of differentiated OLIG2+CC1+ hOLs both at 12 wpg and 20 wpg (**G**). (**H**,**I**) In the oligodendroglial lineage (OLIG2+STEM+), MD1003 significantly decreases the percentage of CC1- hOPCs but increases the percentage of CC1+ hOLs. Two-way ANOVA followed by Tukey’s multiple comparison test (*n*: 3–6 per groups). * *p <* 0.05, and ** *p <* 0.01. Error bars represent SEMs. WPG: weeks post graft, scale bar: 50 µm.

Immunohistochemical analysis of horizontal brain tissue sections collected from the grafted MBP-deficient shiverer mice and containing the corpus callosum revealed that human cells (STEM101+) were found in the corpus callosum and/or in the fimbria of all grafted mice. Of the grafted human cells (STEM101+), 20 to 30% differentiated into OLIG2+ cells (Figure 4). The percentages of human oligodendroglia tended to increase slightly over time (not significant), with no significant difference between the vehicle- (17.79 ± 3.76% and 26.60 ± 2.60% for 12 and 20 wpg, respectively) and MD1003- (26.70 ± 0.90% and 32.91 ± 5.58% for 12 and 20 wpg, respectively) treated groups (Figure 4A–E). Grafted human cells remained in part immature hOPCs (STEM+OLIG2+CC1-) with, for the vehicle groups: 20.72 ± 1.80% and 11.71 ± 3.34% at 12 wpg and 20 wpg, respectively; and for the MD1003 groups: 17.45 ± 3.66% and 13.07 ± 2.64% for 12 wpg and 20 wpg, respectively (Figure 4F). They also differentiated into mature hOLs (STEM+OLIG2+CC1+) with 2.67 ± 0.78% and 9.16 ± 2.29% for 12 wpg and 20 wpg of the vehicle-treated groups; and with 14.98 ± 3.49% and 19.84 ± 4.01% for 12 wpg and 20 wpg of the MD1003-treated groups, respectively (Figure 4G). As expected, the percentage of immature hOPCs had the tendency to decrease with time, while that of mature hOLs increased slightly over time. Further analysis showed a significant increase in the differentiation of hOPCs into CC1+ expressing cells in the MD1003-treated mice over the vehicle-treated ones at both time points, with an average 6-fold increase at 12 wpg (*p* value = 0.047), and 2-fold increase at 20 wpg (*p* value = 0.02). This difference was mainly due to an increase (4.5-fold) in the percentage of mature OLs (CC1+) in the grafted human oligodendroglial population (STEM+OLIG2+) at 12 wpg with 13.33 ± 3.85% vs. 58.68 ± 9.78% (*p* value = 0.0095) for vehicle- and MD1003-treated groups, respectively. In other words, 86.66 ± 3.85% of STEM+OLIG2+ cells were CC1- cells in the vehicle as compared to the MD1003-treated (41.31 ± 9.7%) groups 12 wpg (*p* value = 0.0095). The percentage of CC1+OLIG2+ mature hOLs over total STEM+OLIG2+ human oligodendroglia had a tendency to increase at 20 wpg with 37.45 ± 8.8% vs. 62.31 ± 6.63%, respectively, for the vehicle- and MD1003-treated groups (*p* value = 0.08, Figure 4H,I).

**Figure 5:**
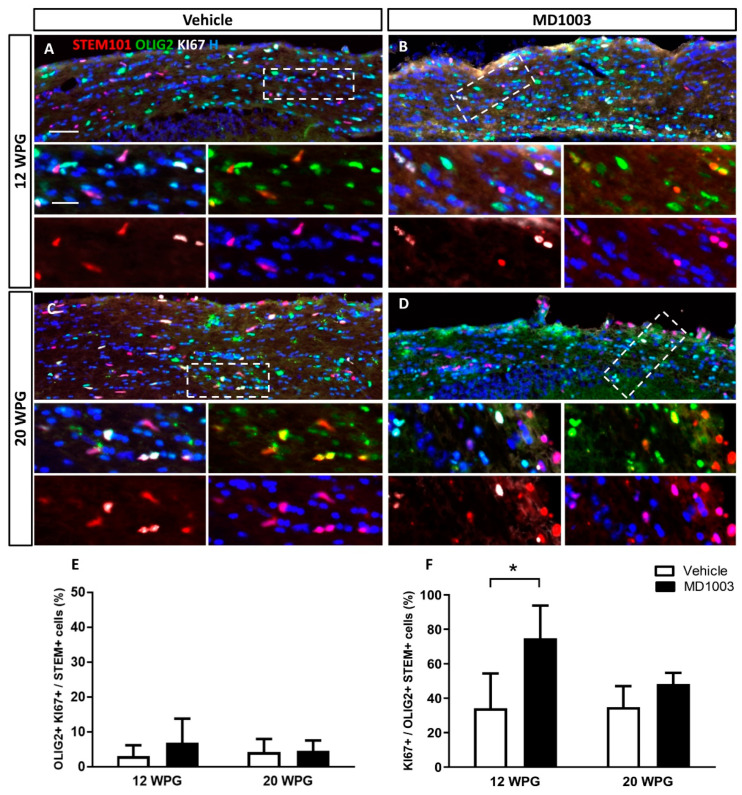
MD1003 increases the percentage of proliferating human oligodendroglial cells. (**A**–**D**) Immunohistochemistry for STEM101+ human cells (red), OLIG2+ oligodendroglia (green) and KI67+ proliferating cell (white) are shown for all the grafted animals at 12 wpg and 20 wpg, treated with vehicle or MD1003. Insets show higher magnification of white dotted boxes in (**A**–**D**). (**E**) The percentage of OLIG2+KI67+ proliferating human cells tends to slightly increase (not significant) in MD1003-treated group at 12 wpg. (**F**) MD1003 significantly increases the percentage of STEM+OLIG2+KI67+ proliferating human oligodendroglia over OLIG2+STEM+ human oligodendroglia. Two-way ANOVA followed by Tukey’s multiple comparison test (*n*: 3–6 per groups). * *p <* 0.05. Error bars represent SEMs. WPG: weeks post graft, scale bar in (**A**–**D**): 50 µm and in the insets: 20 µm.

To better understand if this effect on OPC differentiation into OLs is due to the effect of MD1003 on the grafted cell survival or proliferation rate, tissues were stained for the apoptotic marker, Caspase3 marker, or proliferation marker, KI67. Data did not reveal an effect on the survival of the grafted cells as very few Caspase3+ cells were found in the MD1003- or vehicle-treated groups at the different time points (<0.1%). Moreover, there was no significant differences in proliferation between vehicle- and MD1003-treated groups at 12 and 20 wpg (Figure 5A–E) with 2.99 ± 1.57% vs. 6.8 ± 3.1% of human cells (STEM+) co-expressing OLIG2 and KI67, respectively, for vehicle- vs. MD1003-treated groups at 12 wpg, and 4.13 ± 2.2% vs. 4.51 ± 1.49% of them, at 20 wpg. However, investigating the oligodendroglial population (STEM+OLIG2+) further, revealed a significant 2-fold increase in the percentage of proliferating human oligodendroglial cells (KI67+OLIG2+) (vehicle: 34.19 ± 10.10% vs. MD1003: 74.81 ± 10.97% at 12 wpg (*p* value = 0.017) and a tendency to increase proliferation at 20 wpg (vehicle: 34.86 ± 5.43% vs. MD1003: 48.25 ± 3.22%) (Figure 5F).

**Figure 6:**
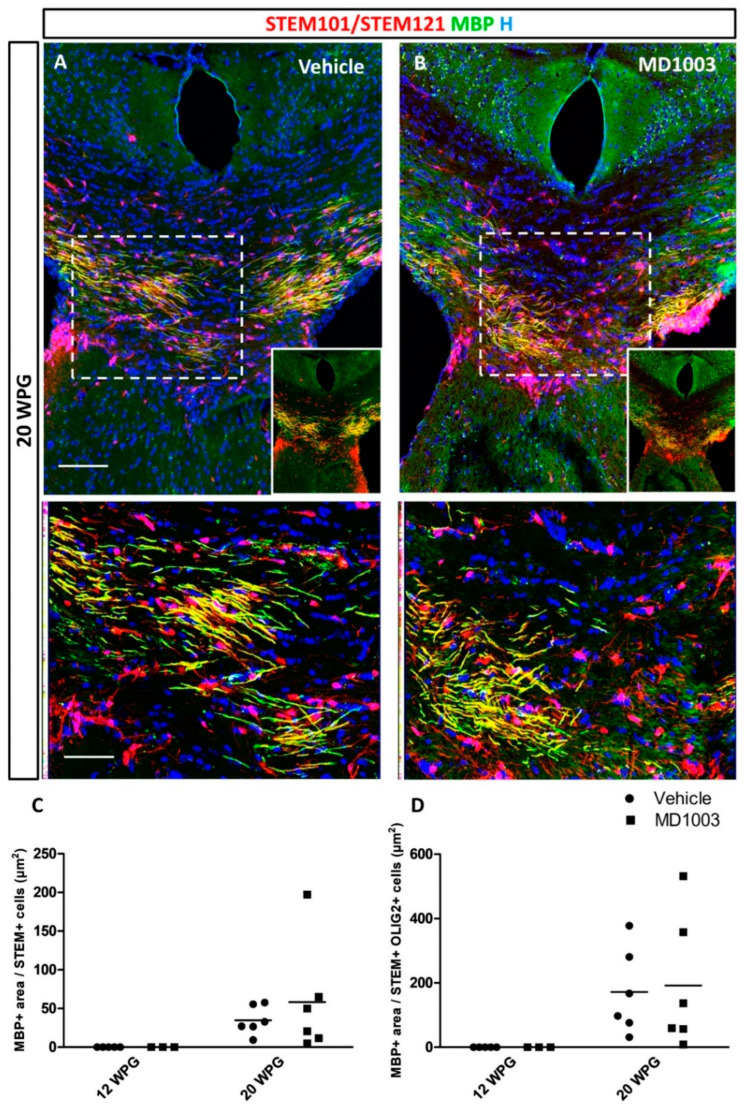
MD1003 does not increase the myelination potential of the hOPCs in vivo. (**A**) hOPCs (STEM101/STEM121 in red), although starting to differentiate as early as 12 wpg (see Figure 4), do not mature into MBP expressing cells (green) at this time point. (**A**,**B**) At 20 wpg they give rise to MBP+ mature oligodendrocytes (higher magnification of dotted boxes in the lower panel insets). MD1003 does not seem to increase the myelination potential of the grafted cells whether MBP is reported per total STEM+ cells (**C**), or total STEM+OLIG2+ OLs (**D**). Two-way ANOVA followed by Tukey’s multiple comparison (*n*: 3–6 per groups). STEM101 (human nuclei marker); STEM121 (human cytoplasmic marker); WPG: weeks post graft, scale bars: 50 µm.

Next, we asked whether the myelination potential of the human cells was affected by MD1003 treatment. MBP immunostaining brain tissue sections collected from the grafted MBP-deficient shiverer mice showed that MBP+ myelin derived from hOLs was absent at 12 wpg, indicating that cells did not mature enough into myelin-forming cells at that age, even in the presence of MD1003. However, MBP+ donor-derived myelin was found at 20 wpg, in all mice of the vehicle- (*n* = 6), and the MD1003-treated groups (*n* = 6) (Figure 6A,B). Immunostaining for STEM and MBP showed several hOLs associated with MBP+ myelin-like segments (insets in Figure 6A,B). Evaluation of the MBP+ area per STEM+ cells (Figure 6C) showed a tendency for MD1003 to enhance myelination. However, this difference was not significant (vehicle group: 34.75 ± 7.56 µm^2^ vs. MD1003 group: 58.12 ± 29.35 µm^2^, *p* value = 0.92). Similar data were observed when the MBP+ area was reported per STEM+OLIG2+ total oligodendroglia (from the immediate adjacent section) (vehicle group: 171.72 ± 54.38 µm^2^; MD1003 group: 191.70 ± 84.72 µm^2^, *p* value = 0.994) (Figure 6D).

**Figure 7:**
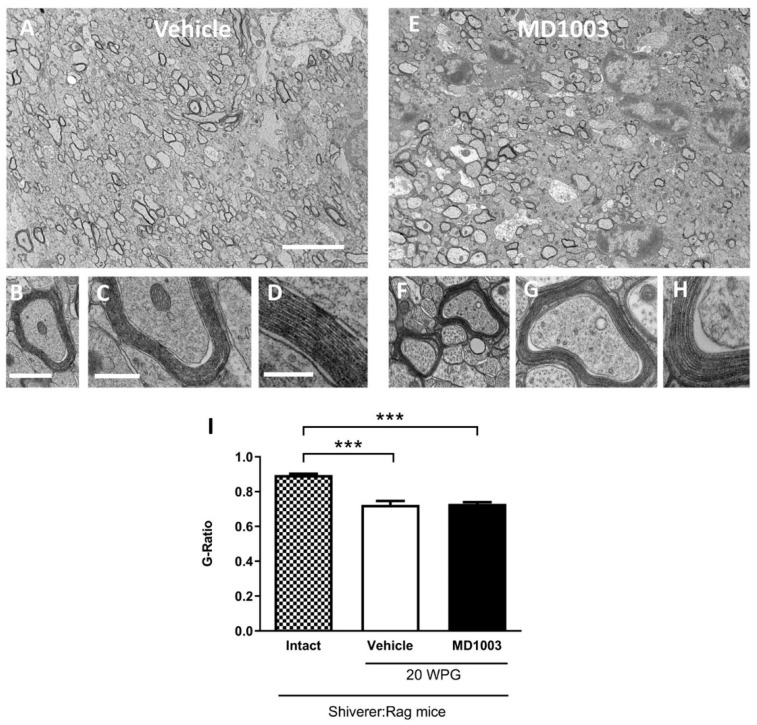
MD1003 does not affect the g-ratio of the human graft-derived myelin. Ultrastructure of graft-derived myelin in sagittal sections of the core of the corpus callosum of shiverer mice at 20 wpg and with vehicle (**A**–**D**) and MD1003 (**E**–**H**) treatments. (**A**,**E**) General views illustrating the presence of some donor-derived electron dense myelin. (**B**,**D**,**F**–**H**) Higher magnifications of axons ensheathed by donor-derived myelin for vehicle- and MD1003-treated groups validate that host axons are surrounded by thick and compact donor-derived myelin. Enlargements in (**D**,**H**), show the presence of the major dense line validating the presence of donor wild-type myelin. No difference in compaction and structure is observed between the myelin of human cells in vehicle-and MD1003-treated mice. (**I**) Quantification of g-ratio revealed significant differences between the graft-derived myelin and intact (not treated and not grafted) shiverer myelin. No significant difference between myelin thickness of axons myelinated by grafted cells in vehicle vs. MD1003. Two-way ANOVA followed by Tukey’s multiple comparison test (*n* = 3–4 mice per group). WPG: week post-graft. *** *p <* 0.001. Error bars represent SEMs. Scale bars in (**A**,**E**): 5 μm, in (**B**,**F**): 1 µm, in (**C**,**G**): 500 nm, in (**D**,**H**): 200 nm.

The fact that MBP-deficient myelin is uncompacted compared to wild-type myelin [33], allowed investigating of the effect of MD1003 on the graft-derived myelin (compacted) by electron microscopy. Compact myelin was found at 20 wpg in the corpus callosum of all grafted shiverer mice (Figure 7A–H). MD1003 had no effect on the percentage of total axon myelination (with 67.77 ± 3.74% and 70.57 ± 2.74% for vehicle- and MD1003-treated groups, respectively) nor the percentage of axons myelinated by the grafted hOLs (with 12.11 ± 1.44% and 16.09 ± 2.55% for vehicle- and MD1003-treated groups, respectively). Since immunohistochemistry for MBP and electron microscopy did not show a significant difference in the myelination potential of the MD1003 group, we investigated a potential effect on the thickness of donor-derived myelin. As expected, we found a significant decrease in the g-ratio (increase in myelin thickness) for grafted animals in both the vehicle- (0.724, *p* value = 0.0001) and MD1003- (0.729, *p* value = 0.0002) treated groups as compared to intact (not treated and not grafted) ones (0.895). However, there was no significant difference in g-ratio between the two grafted groups (*p* value = 0.9622), suggesting that MD1003 did not modulate the graft-derived myelin thickness (Figure 7I).

## 3. Discussion

Studies suggest a strong correlation between metabolic changes, demyelination and neuronal loss [39]. Hence, metabolically active compounds can be beneficial in CNS demyelinating diseases [22]. Lohr et al. (2020) reported that carboxylase biotin levels are reduced in mammalian tauopathies, including brains of human Alzheimer’s disease patients, and biotin rescues mitochondrial dysfunction and neurotoxicity in a tauopathy model [5]. Another study suggests that high dose biotin (MD1003) can regulate impaired metabolism and restore axonal health in an animal model of adrenoleukodystrophy [27]. Biotin-dependent carboxylases are expressed in CNS cells and are found in purified myelin [40], suggesting that these enzymes may be key regulators in oligodendrocyte functions. To address the potential benefit of biotin on myelin, we studied the effect of MD1003 on the behavior of murine or grafted human oligodendroglia in vivo in the dysmyelinating shiverer mouse model.

MD1003 treatment started at P0 via food intake by mothers and continued until the end of study. There is little data on the excretion of biotin into the milk in animals. To ensure MD1003 exposure of the grafted cells before weaning, we measured the MD1003 levels in the plasma and the brains of P14 pups and their mothers. The biodistribution data showed that biotin concentrations both in the plasma and the brains of mice at P14 were as high as those for their mothers (14–16 weeks of age) suggesting that MD1003 could be transmitted through the milk, and that pups were exposed to MD1003 before weaning, reaching brain levels at least 20-fold higher than endogenous levels. The present data are in agreement with those found in the literature for dairy mammals showing that biotin administered orally to cows is transported into the milk and that the concentration is dependent on the dose administered [41].

Our data show that MD1003 increased the percentage of total murine differentiated OLs in the corpus callosum of adult shiverer mice. Although it did not have a significant effect on the percentage of myelinated axons, it increased the percentage of OPCs, early at 12 weeks post treatment and the percentage of mature OLs at 20 weeks post treatment. Cui and colleagues reported that, in vitro, MD1003 did not increase the percentage of rat OPCs but increased the percentage of ensheathing O4+ oligodendrocytes in association with ATP production; yet, it failed to increase the percentage of MBP+ myelinating OLs three days following treatment [28]. Biotin also has the potential to attenuate murine OLs oxidative stress, mitochondria dysfunction and cell death [24]. Moreover, PLP and MBP expression were not, or only slightly affected under treatment with 10–100 ng biotin [24]. These data together with our observations, suggest that MD1003 can enhance the differentiation potential of OPCs (likely providing metabolic support and favoring mitochondrial function) but not myelination. Due to the high metabolic demand of oligodendrocytes to myelinate, it might not be sufficient for the final differentiation of mature oligodendrocytes into myelin-forming cells. It should be underlined, that myelination was followed up to 16/20 wpt, 20 weeks being the survival limit of shiverer mice. If longer survival times had been available, the possibility remains that later time-point analysis would have revealed some differences between the two experimental groups (vehicle and MD1003).

The initial clinical data on treatment with MD1003 showed that, compared with the placebo, MD1003 improved disability outcomes over 12 months (with confirmation at month 15) in patients with progressive MS, with clinical, brain and cervical spinal cord volume improvement [30,31]. However, these data could not be replicated in the SPI2 study [32]. As mentioned above, there have been several studies highlighting the beneficial effects of MD1003 on rodent OPCs. However, to the best of our knowledge, the direct effect of MD1003 on human oligodendroglia in vivo remained elusive. Here, we exploited a humanized dysmyelinating mouse model consisting in grafting human OPCs in the corpus callosum of P1 immune-deficient shiverer mice. Such humanized mouse model of myelin deficiency for drug screening was first used by Abiraman and colleagues in 2015 [42]. The authors showed that systemic treatment with solifenacin, a muscarinic receptor antagonist, can increase the differentiation and myelination potential of human OPCs grafted in the brain of immune-deficient shiverer mice [42]. We used a similar humanized mouse model to investigate the effect of MD1003 on grafted human OPCs.

Our data clearly show that both mouse and human OPCs responded to MD1003 as far as their differentiation into OL is concerned. Yet, they seemed to differ in terms of their proliferative response, with no effect on mouse OPCs, but a clear promoting effect on human OPCs. The effect on human OPC proliferation is in line with previous observations indicating that proliferation persists for a much longer time in the post-natal human brain, or in mouse–human grafting paradigms [18,19]. By contrast, the lack of effect on mouse OPC proliferation is likely due to the fact that murine OPC proliferation occurs much earlier than 12/20 weeks, in the post-natal murine brain [18,43] and was thus no longer detectable when tested in the present paradigm. In spite of promoting differentiation in both species, both immunohistochemistry and electron microscopy showed that MD1003 did not enhance murine and human myelination. While the delayed cell cycle exit of hOPC may have slowed down their myelination process, it would not be the case for murine OPCs since they resume their proliferation within the first postnatal month [44,45].

In the present study, experiments were performed in the shiverer paradigm as they are the optimal recipients to study myelination by exogenous wild-type cells. However, shiverer mice display an increased slow axonal transport rate, and altered neurofilament composition [46] that becomes progressively more prominent, leading to more seizures and a shortened life-span and death at about 20–22 weeks of age [47]. Therefore, it is plausible that although the percentage of differentiated (CC1+Olig2+) murine [43] and human OLs are increasing over time (in a compensatory manner), the overall myelinated axons are not increasing (16 vs. 20 w) as functional axon-myelin units require the continuous incorporation of new compact myelin membranes [48].

As far as exogenous myelination is concerned, MD1003 did not promote the percentage of total myelinated axons in the grafted animals at 20 wpg, nor the percentage of axons myelinated by the human cells, thus inferring the absence of a promoting effect on endogenous myelination by MD1003 in the grafted animals, at this later time-point. Interestingly, the total percentage of myelinated axons, whether treated or not with MD1003, did not overpass that of endogenous myelin of non-grafted animals at 16 wpg, reflecting most-likely, the known competition existing between grafted human and murine OLs [49] and highlighting the inability of MD1003 to modulate this competition. The lack of promoting effect on myelination by human cells could explain why MD1003 failed in the SPI2 clinical trial study [32].

The multi-stage process of oligodendrocyte development is tightly regulated to ensure proper lineage progression of OPCs to mature myelin-producing oligodendrocytes. This process involves complex interactions between several intrinsic signaling pathways that are modulated by an array of extrinsic factors both at the level of OLs and individual axons [50]. Although some factors might regulate the process of oligodendrocyte differentiation, others are required to support their final maturation into myelin-forming cell or myelin compaction [51,52]. It is therefore possible that MD1003 alone was not sufficient to impact on myelination. When murine OLs (with mitochondrial defect and metabolic dysfunction) were exposed to biotin plus α-tocopherol (a known cytoprotective drug) in vitro, MBP expression was strongly enhanced (as compared to biotin or α-tocopherol treatment alone) [24] which suggests that combination therapies targeting can be beneficial to promote myelin repair.

Altogether, our data show that although MD1003 did not increase the myelination potential of murine or human OLs in vivo, it increased the percentage of both murine and human mature oligodendrocytes. In this regard, combination therapy of MD1003 with other neuroprotective/promyelinating compounds could be tested to see if this can result in final maturation of the premyelinating oligodendrocytes into fully myelinating cells. While in the present study, MD1003 was tested as a first approach in developmental conditions, combining this humanized mouse model of myelination with induced-demyelination and MD1003 treatment could provide additional information on the potential benefit of this compound in pathophysiological conditions closer to MS. This humanized mouse model will be a valuable tool for preclinical investigation of metabolically active and/or neuroprotective/promyelinating compounds in demyelinating diseases, and serve as a strong translational model before starting clinical phases.

## 4. Materials and Methods

### 4.1. Mouse Line

Shiverer (C3H strain) mice [37] were crossed with *Rag2^−/−^* (*C3H* strain) null immunodeficient mice [53] to generate *Shi/Shi:Rag2^−/−^* myelin-deficient and immune-deficient mice [38]. Shiverer (*Shi/Shi*) is an autosomal recessive mouse mutation (in myelin basic protein gene) that produces a shivering phenotype associated with CNS hypomyelination in affected mice. They exhibit tonic seizure behavior after weaning and die prematurely, typically between 17 and 20 weeks after birth. Knockout mouse models deficient in recombination activation gene 2 (Rag2) fail to generate mature T and B lymphocytes, preventing immune rejection of the grafted human cells. Mice (in total 37 males and 31 females) were housed under standard conditions of 12-h light/12-h dark cycles with ad libitum access to dry food and water cycle at the ICM institute’s animal facility. Experiments were performed according to European Community regulations and INSERM ethical committee (authorization 75-348; 20 April 2005) and were approved by the local Darwin ethical committee.

### 4.2. hNPC Culture

Human fetuses were obtained after legal abortion at Robert Debré Hospital, Paris 75019, according to the recommendations of the Agence de la Biomedecine (agreement #003187) with patient’s written consent. hNPCs were isolated from lateral ganglionic eminences of human fetuses (7–9 weeks of gestation) in the laboratory of Dr. Anne Baron-Van Evercooren, as previously described [35,36]. Briefly, hNPCs were amplified in spheres in N medium (consisting of a 1:1 mixture of Dulbecco’s modified Eagle’s medium (DMEM)-F12 supplemented with 1% N2 supplement; 0.5% B27; 5 mM HEPES (all Life Technologies, Villebon-sur-Yvette, France); 20 ng/mL insulin (Eurogenetec, Angers, France); 6 mg/mL glucose (Sigma-Aldrich, Saint-Quentin Fallavier, France) supplemented with 20 ng/mL basic fibroblast growth factor (bFGF) and 20 ng/mL epidermal growth factor (EGF) (Peprotech, Neuilly-sur-Seine, France).

### 4.3. Differentiation into hOPC

Five hNPC lines (P5–P8) (RD190, RD191, RD192, RD194 and RD208) were selected for OPC differentiation according to their expansion speed and bipolar cellular morphology. Fetal hNPCs were induced to differentiate into hOPCs after seeding onto polyornithine/laminin-coated dishes, and feeding during 3 weeks with N medium, supplemented with platelet-derived growth factor (PDGF)-AA (10 ng/mL) (Sigma-Aldrich), bFGF and EGF (20 ng/mL). Medium was changed every 2–3 days as previously described [36].

### 4.4. Characterization of the Hopc Cell Lines

The selected hOPC lines were analyzed by immunocytochemistry for their expression of oligodendroglial markers. Cells were prefixed with 4% paraformaldehyde for 10 min at room temperature and washed three times with PBS. Fixed cells were permeabilized by adding 0.2% Triton X-100 (Sigma) in PBS for 15 min at room temperature (this step was omitted for NG2, and O4 staining). Subsequently, a blocking step was performed by incubating the cells with 4% bovine serum albumin (Sigma-Aldrich A7906) for 30 min at room temperature. Primary antibodies were applied overnight at 4 °C in blocking solution. Following three washing steps with PBS, cells were incubated with Alexa Fluor-conjugated secondary antibodies diluted in PBS for 1 h at room temperature. Hoechst dye (1 µg/mL, Sigma-Aldrich) was added to the secondary antibody solutions to stain all cell nuclei. Cells on glass coverslips were mounted in Dako Fluorescent Mounting Medium (Dako) and visualized on a Carl Zeiss microscope equipped with ApoTome2. Primary antibodies used in this study are listed in Table 2.

### 4.5. In Vivo Cell Transplantation

hOPCs derived from fetal hNPC [35,36] were grafted in the corpus callosum of P1–P2 *Shi/Shi:Rag2^−/−^* mice to allow human oligodendrocytes to differentiate and myelinate the shiverer host axons during post-natal development. Briefly, newborn pups were anesthetized on ice for 1–2 min before bilateral injections (10^5^ hOPCS per microliter, 1 µL per injection) rostral to the corpus callosum, 1 mm caudally, 1 mm laterally from bregma and at a depth of 1 mm [20,36].

### 4.6. Treatment by MD1003

All the grafted or non-grafted mice had ad libitum access to pellets: either a standard chow diet (vehicle) or a custom MD1003-supplemented diet provided by the supplier (Ssniff Spezialdiäten GmbH, Ferdinand-Gabriel-Weg 16, 59494 Soest, Germany) from birth up to 12, 16 and 20 weeks of age.

The dose level was selected in agreement with the sponsor (MedDay Pharmaceuticals). The daily dose corresponds to the dose in mice equivalent to the human therapeutic dose (5 mg/kg/day) extrapolated based on body surface area using the conversion table of the Food and Drug Administration (FDA 2005).

The dose of MD1003 in MD1003-supplemented pellets was determined as follows:

- Single daily dose (SD) = 60 mg MD1003/kg/day
- Body weight (BW) = 24 g/animal
- Daily food intake (FI) = 4 g diet/day
- Diet dose (DD) = 360 mg MD1003/kg diet

Each animal was observed once a day, at approximately the same time during the study (excluding weekends and public holidays) for mortality, morbidity and clinical signs. Clinical signs included decreased activity level, limited grooming, abnormal gait, difficulty in rearing, hind limb paralysis, convulsions and tremors. Attention was paid to humane endpoints. If one or more of these humane endpoints was to be observed, then the study director would have decided on the appropriate action (e.g., increased frequency of clinical observations, medical care, wash-out period, euthanasia), after consultation with a veterinarian if necessary. Body weight of each animal was recorded on the day of euthanasia.

### 4.7. Exposure to MD1003

The dose formulations were administered mixed in the diet to the mothers and directly in pellets after weaning. Here we assessed the level of MD1003 in the brain and plasma of P14 pups and mothers following mother feeding, and documented pups’ exposure to MD1003.

Tissue (plasma and brain) collection procedures was initiated after animals were euthanized using Euthasol (sodium pentobarbital) under deep anesthesia and unresponsive to all stimuli:

Plasma: Venous blood (approximately 100 µL from the P14 pups and 150–200 µL from the mother) was collected from direct cardiac puncture (of the right ventricle) into a tube containing lithium heparin and placed on wet ice pending centrifugation. Blood was centrifuged at 3000× *g* for 10 min under refrigerated conditions (set to maintain +4 °C). The plasma was divided into 2 aliquots according to the collected amount (1 aliquot with at least 50 μL and the second, with the remaining volume).

Brain: Right and left hemispheres of the P14 pups and their mothers were rapidly collected into separate cryotubes (prelabeled) after blood sampling, and immersed into liquid nitrogen.

All samples were kept at −80 °C, until analysis by CiToxLAB France using LC-MS/MS.

### 4.8. Immunohistochemistry

For immunohistochemistry, mice were sacrificed by trans-cardiac perfusion-fixation with 4% paraformaldehyde in PBS 1%, and brains were collected and embedded into Shandon Cryomatrix frozen embedding resin (Thermo Scientific 67-690-06, Runcorn, Cheshire WA7 1TA, UK). Frozen brains from 12 and 16 weeks post-treated (grafted or non-grafted) animals were sectioned horizontally at 12 µm thickness with a cryostat (CM3050S; Leica, Nussloch, Germany). Sections were collected from ventral to dorsal in 3 series (A, B, C) of 15 slides each, 12 µm thickness, and 6–8 sections per slide were deposited. Frozen sections were stored at −20 °C until use for immunohistochemistry.

Tissues were permeabilized by adding 0.2% Triton X-100 (Sigma) in PBS for 15 min at room temperature. Subsequently, blocking was performed by incubating sections with 4% bovine serum albumin (Sigma-Aldrich A7906) for 30 min at room temperature. Primary antibodies were applied overnight at 4 °C in blocking solution. Following three washing steps with PBS, tissues were incubated with Alexa Fluor-conjugated secondary antibodies diluted in PBS for 1 h at room temperature. For MBP staining, sections were pretreated with ethanol (10 min, RT).

Secondary antibodies conjugated with FITC, TRITC (SouthernBiotech, Birmingham, AB, USA) or Alexa Fluor 647 (Life Technologies, Carlsbad, CA, USA) were used, respectively, at 1:100 and 1:1000 dilutions. Nuclei were stained with Hoechst (1 µg/mL, Sigma-Aldrich, St. Louis, MO, USA) (1:1000). Sections were mounted in Dako Fluorescent Mounting Medium (Dako, Santa Clara, CA, USA).

In vivo characterization of grafted cells was performed by immunostaining using the following antibodies: human cytoplasmic marker (STEM121) and human nuclei marker (STEM 101) to detect human cells; oligodendroglial markers (OLIG2, CC1) to evaluate differentiation; proliferation marker (KI67); apoptotic marker (Caspase 3) to evaluate oligodendrocyte proliferation and survival; and myelination marker (MBP) to evaluate myelination. The antibodies used are listed in the Table 3.

Tissue scanning, cell visualization and imaging were performed on a Carl Zeiss microscope (Oberkochen, Germany) equipped with ApoTome 2. The MBP staining was done on the immediately adjacent sections to those used for the CC1/OLIG2 staining.

### 4.9. Electron Microscopy

Grafted mice and non-grafted mice were perfused with 1% PBS followed by a mixture of 4% paraformaldehyde/5% glutaraldehyde (Electron Microscopy Sciences, Hatfield, PA, USA) in 1% PBS. After 2-h post-fixation in the same solution, 100-μm-thick sagittal sections were cut and fixed in 2% osmium tetroxide (Sigma-Aldrich) overnight. After dehydration, samples were flat-embedded in Epon. Ultra-thin sections (80 nm) of the median corpus callosum were examined and imaged with a HITACHI 120 kV HT-7700 electron microscope (Krefeld, Germany).

### 4.10. Quantification

#### 4.10.1. Cell Characterization In Vitro

Each staining was performed on duplicate glass coverslips in 3 independent experiments. For each staining, cells were counted from 10 independent fields. A Zeiss microscope was used for cell visualization. Cell counts were performed using ImageJ software (version 1.52t), analyzed with Prism GraphPad software and expressed as a percentage of total Hoechst+ cell number.

#### 4.10.2. Cell Characterization In Vivo

For each animal, six serial sections at 180-μm intervals were analyzed. Cell counts were expressed as the percentage of total OLIG2+, OLIG2+/CC1+ mature- and OLIG2+CC1- immature oligodendroglia over Hoechst+ (for differentiation potential) and OLIG2+KI67+ over Hoechst+ or total OLIG2+ cells (for proliferation potential) in the core of the corpus callosum.

In the grafted mice, quantification was done on all the images taken in each animal from where the STEM+ cells or the MBP staining were found in the corpus callosum and the fimbria. The number of STEM101+ grafted cells expressing Caspase3, KI67, or OLIG2 and CC1 was quantified at 12 and 20 wpg for vehicle- and MD1003-treated mice according to our previously published method [20]. Cell counts were expressed as the percentage of total STEM101+ cells. Furthermore, to evaluate myelination, the MBP+ area was divided per number of STEM+ cells or STEM+/OLIG2+ oligodendroglia to establish the myelination potential of grafted cells in MD1003- vs. vehicle-treated animals.

#### 4.10.3. Electron Microscopy

The percentage of myelinated axons in the corpus callosum of the MD1003- vs. vehicle-treated mice was evaluated for the axons larger than 1 μm diameter on 25–40 visual fields (magnification 62,000×) per animal at 16 weeks of age in non-treated (intact) and non-grafted (*n* = 3) or at 20 wpg (*n* = 3–4 per group) in the grafted mice.

Shiverer mice have uncompacted and thin myelin [33]. Validation of myelination by human cells performed by imaging myelin compaction (presence of major dense line) in shiverer mice at 220,000× magnification at 20 wpg. Myelin thickness was assessed by g-ratio measurements (diameter of axon divided by diameter of axon + myelin). To this end a minimum of 10 images/animal (*n* = 3) were randomly taken at 62,000× in the medial part of the corpus callosum. The g-ratio in each group was determined as previously described [33]. Briefly, the maximum and minimum diameters of a given axon and the maximum and minimum axon plus myelin sheath diameter were measured with ImageJ software. Data were expressed as the mean of the maximal and minimal values for each axon for the MD1003- vs. vehicle-treaded grafted (*n* = 3–4 mice) or in the intact *Shi/Shi Rag^2−/−^* mice (*n* = 3).

### 4.11. Statistical Analysis

Data are presented as means + SEM. Statistical significance was determined by two-tailed Mann–Whitney U test when comparing two statistical groups, and with one-way or two-way analysis of variance (ANOVA) followed by Tukey’s or Sidak’s multiple comparison tests for multiple groups. Non-normally distributed data were analyzed by the corresponding nonparametric tests. Statistical analyses were carried out using GraphPad Prism 8.2.1 (GraphPad Software Inc., San Diego, CA, United States) and a *p* value of less than 0.05 was considered significant. See the figure captions for the number of animals per group and the test used in each experiment.

## Figures and Tables

**Table 1:** Determination of MD1003 concentration in plasma (ng/mL) and in brains (ng/g) of P14 pups following administration of (A) vehicle and (B) MD1003 food to mothers.

	Treatment Group	Sex	Animal Number	Plasma Concentration (ng/mL)	Brain Concentration (ng/g)
A	**Vehicle**	Male	983	<50	<50
			984	<50	<50
		Female	985	<50	<50
			986	<50	<50
			987	<50	<50
			988	<50	<50
		MOTHER	818	<50	<50
B	**MD1003**	Male	972	2542	1032
			973	2750	1088
			974	3093	1257
		Female	975	3940	1523
			976	3155	1274
			977	3008	1207
			978	2756	1132
		MOTHER	817	2134	859

**Table 2:** List of primary antibodies used for in vitro cell characterization.

Antibody	Dilution	Source
Mouse anti-O4	1:100	R&D (MAB1326)
Rabbit anti-NG2	1:200	Millipore (AB5320)
Goat anti-SOX10	1:50	R&D systems (AF2864)
Rabbit anit-OLIG2	1:400	Millipore (AB910)
Rat anti-PDGFRα	1:200	BD Pharmingen (558774)
Mouse anti-NKX2.2	1:100	Developmental Studies Hybridoma Bank (74.5A5)

**Table 3:** List of primary antibodies used for in vivo cell characterization.

Antibody	Dilution	Source
Human cytoplasmic anti-STEM121	1:100	Takara (Y40410)
Human nucleus anti-STEM 101	1:100	Takara (Y40400)
Rabbit anti-Olig2	1:400	Millipore (AB910)
Rat anti-CC1	1:200	BD Pharmingen (558774)
Rat anti-MBP	1:100	Abcam (ab7349)
Rat anti-KI67	1:100	Santa Cruz, sc-23900
Rabbit anti-Caspase 3	1:100	Cell signaling, 9661

## Data Availability

Not applicable.
